# Secretogranin II influences the assembly and function of MHC class I in melanoma

**DOI:** 10.1186/s40164-023-00387-1

**Published:** 2023-03-11

**Authors:** Tamara Steinfass, Juliane Poelchen, Qian Sun, Giovanni Mastrogiulio, Daniel Novak, Marlene Vierthaler, Sandra Pardo, Aniello Federico, Laura Hüser, Thomas Hielscher, Rafael Carretero, Rienk Offringa, Peter Altevogt, Viktor Umansky, Jochen Utikal

**Affiliations:** 1 Skin Cancer Unit, German Cancer Research Center (DKFZ), INF 280, 69120 Heidelberg, Germany; 2 Department of Dermatology, Venereology and Allergology, University Medical Center Mannheim, Ruprecht Karl University of Heidelberg, Theodor-Kutzer-Ufer 1-3, 68167 Mannheim, Germany; 3 DKFZ-Hector Cancer Institute at the University Medical Center Mannheim, Theodor-Kutzer-Ufer 1-3, 68167 Mannheim, Germany; 4 Faculty of Biosciences, Ruprecht Karl University of Heidelberg, Heidelberg, Germany; 5 Joint Immunotherapeutics Laboratory, German Cancer Research Center (DKFZ), INF 280, 69120 Heidelberg, Germany; 6 Division of Biostatistics, German Cancer Research Center (DKFZ), INF 581, 69120 Heidelberg, Germany; 7 Division of Molecular Oncology of Gastrointestinal Tumors, German Cancer Research Center (DKFZ), INF 280, 69120 Heidelberg, Germany; 8 Department of Surgery, University Hospital Heidelberg, INF 420, 69120 Heidelberg, Germany; 9 Mannheim Institute for Innate Immunoscience (MI3), Medical Faculty Mannheim, Ruprecht Karl University of Heidelberg, Ludolf-Krehl-Straße 13–17, 68167 Mannheim, Germany; 10 Division of Pediatric Neurooncology, German Cancer Research Center (DKFZ), INF 280, 69120 Heidelberg, Germany; 11 Division of Biochemistry and Molecular Biology, Johns Hopkins Bloomberg School of Public Health, 615 N. Wolfe Street, Baltimore, MD 21205 USA

**Keywords:** Melanoma, SCG2, HLA, MHC class I, Prognosis

## Abstract

Melanoma is the deadliest form of skin cancer showing rising incidence over the past years. New insights into the mechanisms of melanoma progression contributed to the development of novel treatment options, such as immunotherapies. However, acquiring resistance to treatment poses a big problem to therapy success. Therefore, understanding the mechanisms underlying resistance could improve therapy efficacy. Correlating expression levels in tissue samples of primary melanoma and metastases revealed that secretogranin 2 (SCG2) is highly expressed in advanced melanoma patients with poor overall survival (OS) rates. By conducting transcriptional analysis between SCG2-overexpressing (OE) and control melanoma cells, we detected a downregulation of components of the antigen presenting machinery (APM), which is important for the assembly of the MHC class I complex. Flow cytometry analysis revealed a downregulation of surface MHC class I expression on melanoma cells that showed resistance towards the cytotoxic activity of melanoma-specific T cells. IFNγ treatment partially reversed these effects. Based on our findings, we suggest that SCG2 might stimulate mechanisms of immune evasion and therefore be associated with resistance to checkpoint blockade and adoptive immunotherapy.

**Supplementary Information:**

The online version contains supplementary material available at 10.1186/s40164-023-00387-1.

**To the Editor**,

Melanoma is the deadliest skin cancer type and often associated with poor prognosis despite a variety of treatment options [1, 2]. The major histocompatibility complex class I (MHC-I) presents fragments of intracellular peptides on the cell surface to CD8 + T cells [3]. MHC-I, the TAP complex (transporter associated with antigen processing) and chaperones located in the ER constitute the antigen presenting machinery (APM) [4–6]. Impairment of MHC-I assembly could affect the efficiency of immunotherapies relying on activation of CD8 + T cells. SCG2 belongs to the granin family and plays an essential role in secretory granule formation and biogenesis [7, 8]. We showed recently that high SCG2 expression correlates with low survival rate of melanoma patients with metastases [9]. Here, we investigated the role of SCG2 in melanoma and its contribution to immunotherapy resistance.

Analysis of publicly available data of metastatic melanoma patients from DFCI, Nature Medicine 2019 (n = 121; Fig. 1A) [10] revealed that high intratumoral SCG2 expression (log2 SCG2 ≥ 1) correlated with a tendency towards lower OS compared to low intratumoral SCG2 expression (log2 SCG2 < 1; p = 0.0531). Data from a GSE database (GSE7553) [11] confirmed higher SCG2 levels in primary melanoma and metastases compared to normal skin (Fig. 1B) and higher levels of SCG2 in primary melanoma compared to nevi (Fig. 1C). By utilizing cell cycle analysis comparing empty vector (EV) control and ectopically SCG2 OE melanoma cells we ascertained no difference in cell cycle phases between both groups (Fig. 1E).

**Fig. 1:**
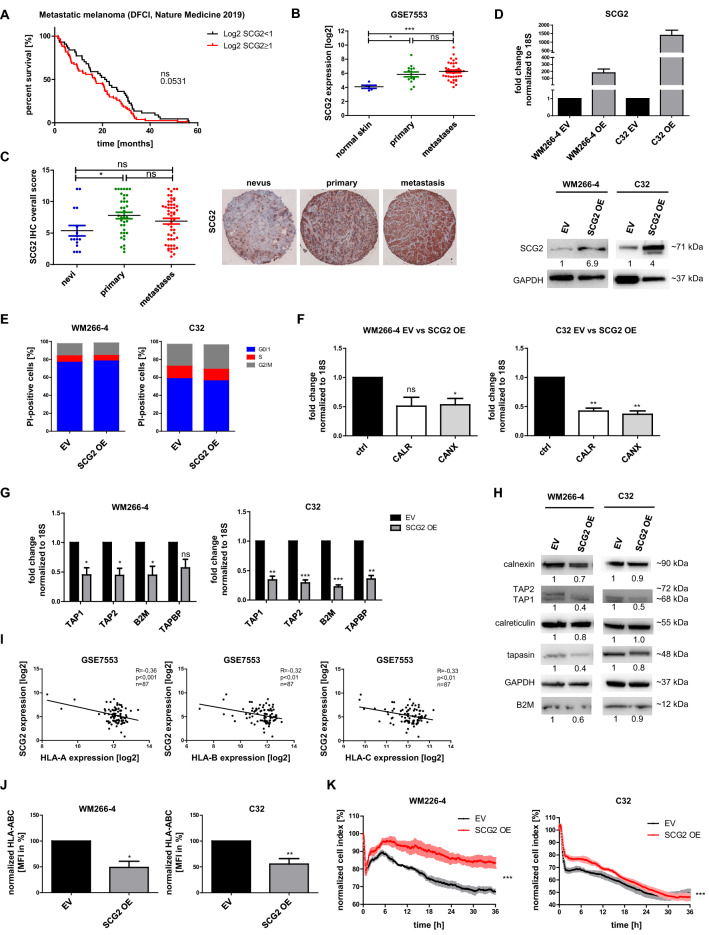
SCG2 is more strongly expressed in melanoma compared to healthy skin and reduces the overall survival (OS) of melanoma patients (**A**) SCG2 expression data from DFCI, Nature Medicine 2019. Kaplan–Meier curve showing OS of patients (n = 121) with metastatic melanoma with high intratumoral SCG2 expression (Log2 SCG2 ≥ 1) compared to patients with low intratumoral SCG2 expression (Log2 SCG2 < 1). **B** Patient data obtained from the GSE7553 database showing SCG2 expression levels as log2 in normal skin (n = 5), primary melanoma (n = 14), and melanoma metastases (n = 40). Statistical analysis was conducted using one-way ANOVA. **C** SCG2 immunohistochemistry (IHC) staining of patient samples from nevi (n = 16), primary melanoma (n = 37), and melanoma metastases (n = 52). Statistical analysis was conducted using one-way ANOVA. (**D**) Confirmation and quantification of SCG2 overexpression (OE) in WM266-4 and C32 melanoma cells on mRNA (upper panel) and protein (lower panel) level. Empty vector (EV) cells were used as a reference. 18S was used as an internal control. GAPDH was used as loading control. Data represent mean ± s.e.m. (n ≥ 3) (**E**) Cell cycle analysis of WM266-4 (left panel) and C32 (right panel) EV and SCG2 OE cells. DNA was stained using propidium iodide (PI) and the number of PI-positive cells was determined using flow cytometry (n = 3). **F** Fold change of mRNA expression levels of the ER markers and APM components calreticulin (CALR) and calnexin (CANX) in WM266-4 (left panel) and C32 (right panel) SCG2 OE cells compared to EV control (ctrl). Data represent mean ± s.e.m. (n ≥ 3). **G** Fold change of mRNA expression of the APM components TAP1, TAP2, B2M, and TAPBP (tapasin) in SCG2 OE cells (WM266-4, left panel, and C32, right panel) compared to EV control. 18S was used as endogenous control. Data represent mean ± s.e.m. (n ≥ 3). **H** Protein levels of the APM components calnexin, TAP2, TAP1, calreticulin, tapasin, and B2M in WM266-4 (left panel) and C32 (right panel) EV and SCG2 OE cells. GAPDH was used as loading control. **I** Correlation of the expression of SCG2 and HLA-A, HLA-B, and HLA-C, respectively, in melanoma patients (n = 87) according to the data from GSE7553.** J** Mean fluorescence intensity (MFI) of HLA-ABC-positive ( +) WM266-4 (left panel) and C32 (right panel) cells comparing SCG2 OE to EV control. Data represent mean ± s.e.m. (n ≥ 3). **K** T cell cytotoxicity assay performed with MART-1-specific T cells measured by xCELLigence RTCA impedance assay. The interaction of the WM266-4 (left panel) and C32 (right panel) cells with the gold biosensors was measured through the cellular impedance. This impedance value is plotted as normalized cell index, which correlates with the cell number. An increase of the normalized cell index indicates cell proliferation while a decrease represents the neutralization of melanoma cells through T cell-mediated cytotoxicity. We compared the normalized cell index of WM266-4 (left panel) and C32 (right panel) EV (black) and SCG2 OE (red) cells over time. Data represent mean ± s.e.m. of three independent experiments (n = 3). *p < 0.05; **p < 0.01; ***p < 0.001; “ns” refers to p ≥ 0.05

Next, microarray gene expression analysis followed by Reactome, KEGG, and gene ontology database analysis demonstrated that pathways involved in antigen presentation through MHC-I were impaired after SCG2 OE (Additional file 1). Additionally, SCG2 OE decreased the expression of several APM components (Fig. 1F–H).

Hereafter, we analyzed the expression of SCG2 and the HLA genes, which encode the heavy chain of the MHC-I complex, in melanoma patients (n = 87) from a GSE database (GSE7553) and found a highly significant negative correlation between SCG2 and HLA-A, HLA-B and HLA-C expression (Fig. 1I). Furthermore, flow cytometry revealed significantly reduced surface presentation of HLA-ABC on SCG2 OE melanoma cells (Fig. 1J). However, the percentage of HLA-ABC-positive cells was not altered (Additional file 2). We then performed a T cell cytotoxicity assay using SCG2 OE cells and cytotoxic T cells specific for melanoma antigen recognized by T cells (MART)-1. Our data indicate that SCG2 OE cells were more resistant to T cell-induced cytotoxicity compared to EV control cells (Fig. 1K, Additional file 3).

Next, we treated SCG2 OE cells with IFNγ, which enhances MHC-I expression through the activation of the Stat1-pathway^12^. We observed significant upregulation of HLA-ABC expression on SCG2 OE melanoma cells (Fig. 2A). The percentage of HLA-ABC-positive cells remained unchanged (Additional file 4). Quantification of STAT1 mRNA expression levels showed significant downregulation upon SCG2 OE. However, IFNγ treatment increased STAT1 mRNA expression in EV and SCG2 OE cell lines (Fig. 2B). Western blot analysis demonstrated increased total Stat1 and pStat1 levels after IFNγ treatment in EV and SCG2 OE cells (Fig. 2C). Moreover, we observed a decrease of total Stat1 and pStat1 in untreated SCG2 OE cells.

**Fig. 2:**
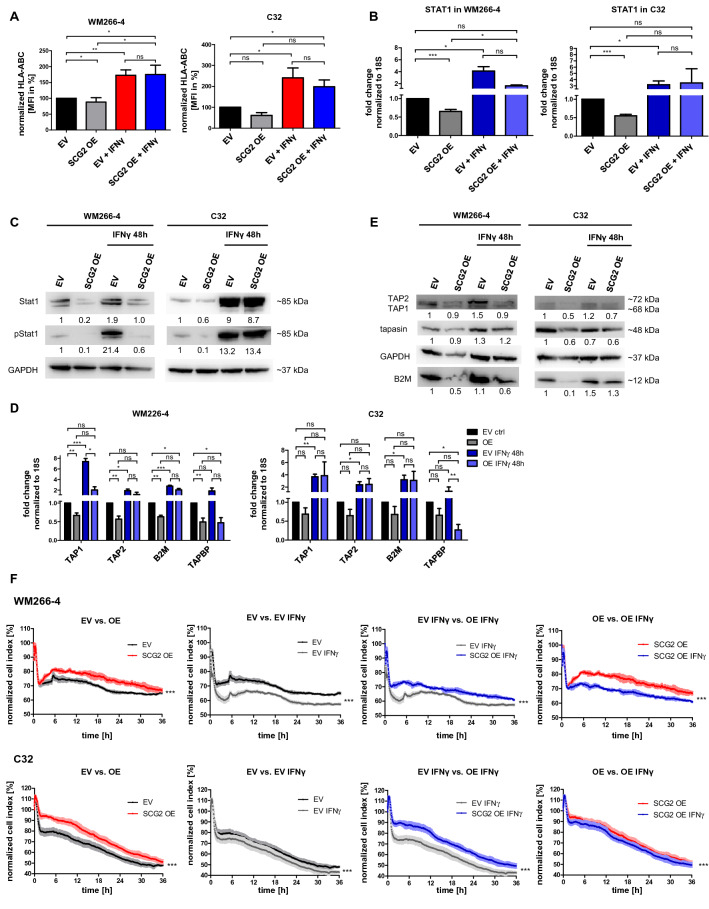
SCG2 OE influences Stat1-induced MHC class I surface presentation, which can be partially restored by IFNγ treatment (**A**) Mean fluorescence intensity (MFI) of HLA-ABC-positive ( +) WM266-4 (left panel) and C32 (right panel) EV and SCG2 OE cells before and after IFNγ treatment (10 ng/ml, 48 h). Data represent mean ± s.e.m. (n ≥ 3). **B** Fold change of Stat1 mRNA expression in WM266-4 (left panel) and C32 (right panel) EV and SCG2 OE cells before and after IFNγ treatment (10 ng/ml, 48 h). 18S was used as endogenous control. Data represent mean ± s.e.m. (n ≥ 3). **C** Western blot analysis of the expression of total Stat1 and pStat1 (phosphorylated Stat1) in WM266-4 (left panel) and C32 (right panel) EV and SCG2 OE cells before and after IFNγ treatment (10 ng/ml, 48 h). GAPDH was used as a loading control. **D** Fold change of mRNA expression levels of the APM components TAP1, TAP2, B2M, and TAPBP (tapasin) in WM266-4 (left panel) and C32 (right panel) EV and SCG2 OE cells before and after IFNγ treatment (10 ng/ml, 48 h). 18S was used as an endogenous control. Data represent mean ± s.e.m. (n ≥ 3). **E** Western blot analysis of the expression of the APM components TAP2, TAP1, tapasin, and B2M in WM266-4 (left panel) and C32 (right panel) EV and SCG2 OE cells before and after IFNγ treatment (10 ng/ml, 48 h). GAPDH was used as a loading control. **F** The upper panel shows impedance value plotted as the normalized cell index of IFNγ-treated and untreated WM266-4 EV and SCG2 OE cells over time. The lower panel shows the normalized cell index of IFNγ-treated and untreated C32 EV and SCG2 OE cells over time. Graphs show comparisons of the normalized cell index between EV and SCG2 OE, EV and EV treated with IFNγ, EV and SCG2 OE both treated with IFNγ, as well as SCG2 OE and SCG2 OE treated with IFNγ. An increase of the normalized cell index represents cell proliferation and a decrease represents the killing of melanoma cells through T cell-mediated cytotoxicity. EV cells are highlighted in black, IFNγ-treated EV cells are highlighted in grey, SCG2 OE cells are highlighted in red, and IFNγ-treated SCG2 OE cells are highlighted in blue. Cells were treated with10 ng/ml IFNγ for 48 h. Data represent mean ± s.e.m. of three independent experiments (n = 3). *p < 0.05; **p < 0.01; ***p < 0.001; “ns” refers to ≥ 0.05

IFNγ treatment also increased TAP1, TAP2, and B2M mRNA expression in EV and SCG2 OE cells (Fig. 2D). Western blot analysis confirmed upregulation of TAP1, TAP2, and B2M (Fig. 2E).

Thereafter, we examined the effect of the IFNγ treatment on the sensitivity of SCG2 OE cells to T cell-mediated cytotoxicity. We detected a significantly higher sensitivity of IFNγ-treated EV and SCG2 OE cells compared to untreated cells (Fig. 2F, Additional files 5, 6). When comparing IFNγ-treated EV and SCG2 OE cells we found that SCG2 OE cells were less sensitive towards T cell-mediated cytotoxicity .

We demonstrate here that high intratumoral SCG2 levels correlated with worse prognosis for melanoma patients. SCG2 OE led to downregulation of APM components, which resulted in decreased MHC-I expression and reduced sensitivity of melanoma cells towards T cell-induced cytotoxicity. IFNγ treatment partially counteracted downregulation of APM components and MHC-I. However, IFNγ-treated SCG2 OE cells were still more resistant to T cell-induced cytotoxicity. Our results contribute to understanding melanoma immune evasion and the role of SCG2 in this process. Therefore, SCG2 could be a valuable prognostic factor, potentially influencing the success of checkpoint blockade and adoptive immunotherapy.

## Supplementary Information

**Additional file 1: Table S1.** KEGG pathway analysis showing pathways predicted to be decreased in WM266-4 and C32 SCG2 OE melanoma cells compared to their control (EV).** Table S2.** Gene ontology pathway analysis showing pathways predicted to be decreased in WM266-4 and C32 SCG2 OE melanoma cells compared to their control (EV).** Table S3.** Reactome pathway analysis showing pathways predicted to be decreased in WM266-4 and C32 SCG2 OE melanoma cells compared to their control (EV).

**Additional file 2: Figure S1.** SCG2 OE does not change the percentage of HLA-ABC-positive cells.

**Additional file 3: Figure S2.** Correlation of high SCG2 expression with decreased MHC class I surface presentation on melanoma cells.

**Additional file 4: Figure S3.** IFNγ treatment does not influence the percentage of HLA-ABC-positive cells or SCG2 expression.

**Additional file 5: Figure S4.** SCG2 OE melanoma cells are more resistant to T cell-mediated cytotoxicity.

**Additional file 6.** Additional materials and methods.

## Data Availability

The raw microarray data generated in this study are available in GEO under accession number GSE203179. Other data that support the findings of this study are available from the corresponding author upon request.

## Abbreviations

APM: Antigen presenting machinery
B2M: β2-Microglobulin
CALR: Gene encoding calreticulin
CANX: Gene encoding calnexin
ER: Endoplasmic reticulum
EV: Empty vector
HC: Heavy chain
HLA: Human leukocyte antigen
IFN: Interferon
MART-1: Melanoma antigen recognized by T cells 1
MHC: Major histocompatibility complex
OE: Overexpression
pStat1: Phospho-Stat1
SCG2: Secretogranin 2
SEM: Standard error of the mean
SN: Secretoneurin
TAPBP: Gene encoding tapasin
TMA: Tissue microarray
