# Supplementary material for: Secretogranin II influences the assembly and function of MHC class I in melanoma
Source: Exp Hematol Oncol. 2023 Mar 11;12:29. doi: 10.1186/s40164-023-00387-1 (PMC10007832; doi:10.1186/s40164-023-00387-1)
Supplement: Supplementary file 1 — Additional file 1: Table S1. KEGG pathway analysis showing pathways predicted to be decreased in WM266-4 and C32 SCG2 OE melanoma cells compared to their control (EV). Table S2. Gene ontology pathway analysis showing pathways predicted to be decreased in WM266-4 and C32 SCG2 OE melanoma cells compared to their control (EV). Table S3. Reactome pathway analysis showing pathways predicted to be decreased in WM266-4 and C32 SCG2 OE melanoma cells compared to their control (EV). [file 40164_2023_387_MOESM1_ESM.docx]

**Additional Tables**

| Pathway | Direction | No. of genes | p-value (WM266-4) | p-value (C32) |
| --- | --- | --- | --- | --- |
| Antigen processing and presentation | Down | 62 | 0.0916 | 0.0001 |

Additional file 1**: S1. KEGG** **pathway analysis showing pathways predicted to be decreased in WM266-4 and C32 SCG2 OE melanoma cells compared to their control (EV).**

| Pathway | Direction | No. of genes | p-value (WM266-4) | p-value (C32) |
| --- | --- | --- | --- | --- |
| antigen processing and presentation of exogenous peptide antigen via MHC class I, TAP-dependent | Down | 74 | 0.0028 | 0.0013 |
| antigen processing and presentation of peptide antigen via MHC class I | Down | 94 | 0.0003 | 0.0014 |
| antigen processing and presentation of exogenous peptide antigen via MHC class I | Down | 77 | 0.0021 | 0.0016 |
| antigen processing and presentation of peptide antigen | Down | 184 | 0.0381 | 0.0058 |
| antigen processing and presentation of exogenous peptide antigen | Down | 171 | 0.0466 | 0.0116 |
| MHC protein complex assembly | Down | 6 | 0.0173 | 0.0050 |

**Additional file 1. S2. Gene ontology pathway analysis showing pathways predicted to be decreased in WM266-4 and C32 SCG2 OE melanoma cells compared to their control (EV).**

| Pathway | Direction | No. of genes | p-value (WM266-4) | p-value (C32) |
| --- | --- | --- | --- | --- |
| Antigen processing: Ubiquitination & Proteasome degradation | Down | 302 | 0.1179 | 0.0450 |
| Class I MHC mediated antigen processing & presentation | Down | 370 | 0.0458 | 0.0749 |
| Antigen Presentation: Folding, assembly and peptide loading of class I MHC | Down | 25 | 0.00001 | 0.7554 |

**Additional file 1: S3. Reactome pathway analysis showing pathways predicted to be decreased in WM266-4 and C32 SCG2 OE melanoma cells compared to their control (EV).**
