# Supplementary material for: Secretogranin II influences the assembly and function of MHC class I in melanoma
Source: Exp Hematol Oncol. 2023 Mar 11;12:29. doi: 10.1186/s40164-023-00387-1 (PMC10007832; doi:10.1186/s40164-023-00387-1)
Supplement: Supplementary file 2 — Additional file 2: Figure S1. SCG2 OE does not change the percentage of HLA-ABC-positive cells. [file 40164_2023_387_MOESM2_ESM.docx]

**Additional file S: figure S1**


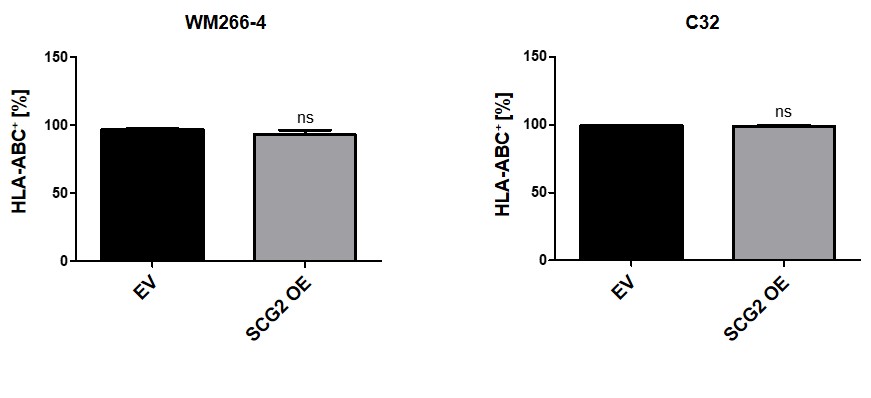


**Additional file 2. Fig. S1. SCG2 OE does not change the percentage of HLA-ABC-positive cells**

Percentage of HLA-ABC-positive (+) WM266-4 (left panel) and C32 (right panel) EV and SCG2 OE cells. “ns” refers to p ≥ 0.05.
