# Supplementary material for: Secretogranin II influences the assembly and function of MHC class I in melanoma
Source: Exp Hematol Oncol. 2023 Mar 11;12:29. doi: 10.1186/s40164-023-00387-1 (PMC10007832; doi:10.1186/s40164-023-00387-1)
Supplement: Supplementary file 3 — Additional file 3: Figure S2. Correlation of high SCG2 expression with decreased MHC class I surface presentation on melanoma cells. [file 40164_2023_387_MOESM3_ESM.docx]

**Additional file 3 figure S2**


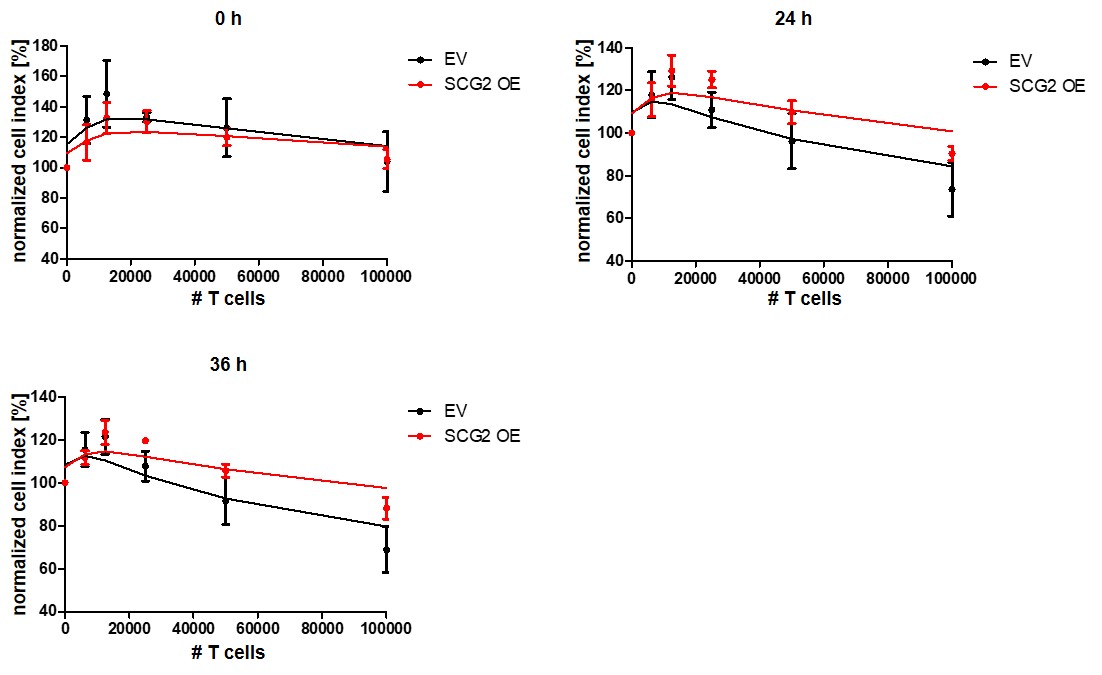


**Additional file 3 Fig.S2. Correlation of high SCG2 expression with decreased MHC class I surface presentation on melanoma cells**

Titration of different amounts of MART-1-specific T cells (0; 6,250; 12,500; 25,000; 50,000; and 100,000) to determine optimal T cell number for a T cell cytotoxicity assay performed using the xCELLigence RTCA impedance assay. Impedance value is plotted as the normalized cell index of WM266-4 EV and SCG2 OE cells at 0h, 24h, and 36h time point. An increase of the normalized cell index represents cell proliferation and a decrease represents the neutralization of melanoma cells through T cell-mediated cytotoxicity. EV cells are highlighted in black, SCG2 OE cells are highlighted in red.
