# Supplementary material for: Secretogranin II influences the assembly and function of MHC class I in melanoma
Source: Exp Hematol Oncol. 2023 Mar 11;12:29. doi: 10.1186/s40164-023-00387-1 (PMC10007832; doi:10.1186/s40164-023-00387-1)
Supplement: Supplementary file 4 — Additional file 4: Figure S3. IFNγ treatment does not influence the percentage of HLA-ABC-positive cells or SCG2 expression. [file 40164_2023_387_MOESM4_ESM.docx]

**Additonal file 4: figure S3**


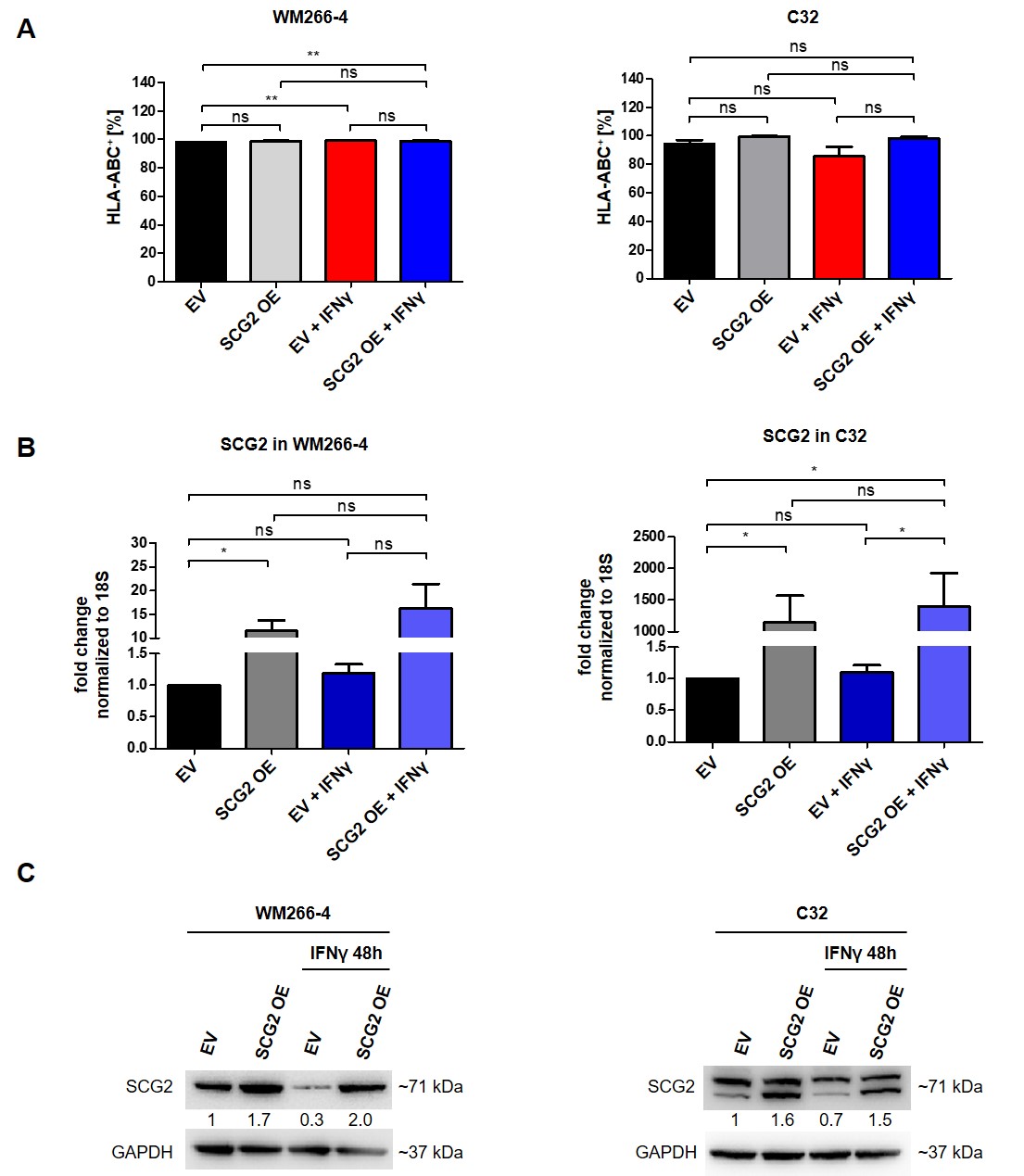


**Additonal file 4: Fig. S3. IFNγ treatment does not influence the percentage of HLA-ABC-positive cells or SCG2 expression**

**(A)** Percentage of HLA-ABC-positive (+) WM266-4 (left panel) and C32 (right panel) EV and SCG2 OE cells before and after IFNγ treatment (10 ng/ml, 48h). **(B)** Fold change of SCG2 mRNA expression in WM266-4 (left panel) and C32 (right panel) EV and SCG2 OE cells before and after IFNγ treatment (10 ng/ml, 48h). 18S was used as endogenous control. **(C)** Western blot analysis of SCG2 expression in WM266-4 (left panel) and C32 (right panel) EV and SCG2 OE cells before and after IFNγ treatment (10 ng/ml, 48h). GAPDH was used as a loading control. *p < 0.05; **p < 0.01; “ns” refers to p ≥ 0.05.
