# Supplementary material for: Secretogranin II influences the assembly and function of MHC class I in melanoma
Source: Exp Hematol Oncol. 2023 Mar 11;12:29. doi: 10.1186/s40164-023-00387-1 (PMC10007832; doi:10.1186/s40164-023-00387-1)
Supplement: Supplementary file 5 — Additional file 5: Figure S4. SCG2 OE melanoma cells are more resistant to T cell-mediated cytotoxicity. [file 40164_2023_387_MOESM5_ESM.docx]

**Additonal file 5: figure 4**


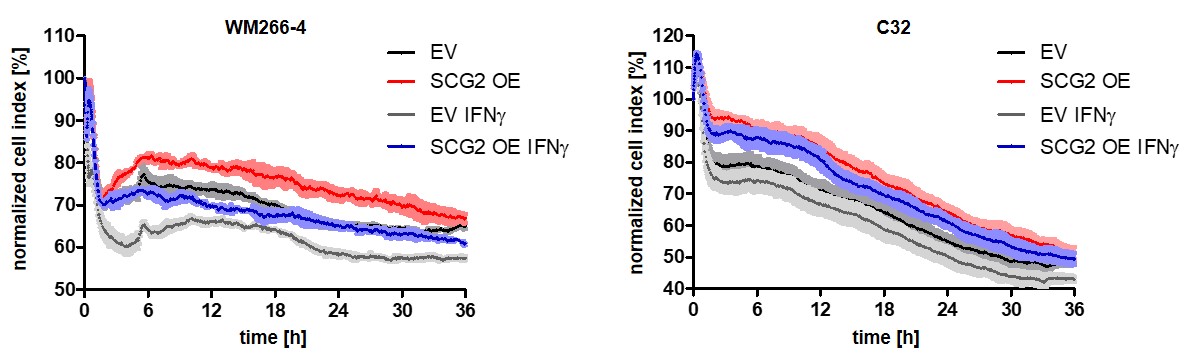


**Additonal file 5: Fig. S4. SCG2 OE melanoma cells are more resistant to T cell-mediated cytotoxicity**

Left side shows impedance value plotted as the normalized cell index of IFNγ-treated and untreated WM266-4 EV and SCG2 OE cells over time. Right side shows the normalized cell index of IFNγ-treated and untreated C32 EV and SCG2 OE cells over time. An increase of the normalized cell index represents cell proliferation and a decrease represents the neutralization of melanoma cells through T cell-mediated cytotoxicity. EV cells are highlighted in black, IFNγ-treated EV cells are highlighted in grey, SCG2 OE cells are highlighted in red, and IFNγ-treated SCG2 OE cells are highlighted in blue. Cells were treated with 10 ng/ml IFNγ for 48h.
