# Supplementary material for: Secretogranin II influences the assembly and function of MHC class I in melanoma
Source: Exp Hematol Oncol. 2023 Mar 11;12:29. doi: 10.1186/s40164-023-00387-1 (PMC10007832; doi:10.1186/s40164-023-00387-1)
Supplement: Supplementary file 6 — Additional file 6. Additional materials and methods. [file 40164_2023_387_MOESM6_ESM.docx]

## Materials and Methods

**Cell lines and compounds**

Melanoma cells WM266-4, C32, and HT144 (all from ATCC, Virginia, USA) were cultured in DMEM medium (Dulbecco-modified essential medium; Life Technologies, Carlsbad, CA, USA) containing 10% FCS (Biochrom, Berlin, Germany), 0.1 mM β-mercaptoethanol (Life Technologies), 1% non-essential amino acids (Sigma-Aldrich, St. Louis, MO, USA), and 1% penicillin/streptomycin (Sigma-Aldrich). Cells were stored in a humidified incubator at 37°C and 5% CO_2_. For IFNγ treatment, cells were exposed to 10 ng/ml IFNγ (Peprotech, Hamburg, Germany) for 48h (retreated after 24h). All experiments were performed when cells reached 50-80% confluency. To exclude a mycoplasma contamination, cells were tested on a regular basis using Venor®GeM Classic Mycoplasma detection kit (Minerva Biolabs, Berlin, Germany).

**Lentiviral transduction**

HEK293T cells were used for lentiviral particle production. For transfection, HEK293T cells were grown to ~60% confluency. Transfection was performed by mixing 11 µg of the plasmid containing the gene/shRNA of interest with the packaging plasmids pCMV-VSV-G (5.5 µg; #8454 Addgene, Watertown, MA, USA) and pCMV-dR8.91 (8.25 µg, Konrad Hochedlinger, Harvard, Boston, MA, USA), and X-treme GENE® (Roche, Basel, Switzerland) solution in pure DMEM. After 30 min incubation at room temperature (RT), the DNA X-treme GENE® mixture was added to the HEK293T cells and 12h later, the supernatant was discarded. After 24, 36, and 48h, the virus particle containing supernatant was collected and sterile filtered using a 0.45 mm PVDF filter (Carl Roth, Karlsruhe, Germany). To infect the melanoma cells, the filtered virus suspension was added to the cells for 24h. To increase the transduction efficiency 8 µg/ml polybrene (Sigma-Aldrich) were added. After 24h, cells were re-infected with the same virus in fresh medium without adding polybrene. 48h after the first transduction, cells were washed with PBS and cultured in normal culturing medium. To remove the non-transduced cells, cells were selected for 3 days in medium containing 0.5-1 µg/ml puromycin (Carl Roth) or 10-15 µg/ml blasticidine (Sigma-Aldrich). The following plasmids were used: pLEX980-hSCG2 for SCG2 OE and the respective empty vector (EV) control. Both plasmids were derived from pLEX980-SETDB1 obtained from Craig Ceol (Children’s Hospital Boston, MA, USA). The EV was generated by removing SETDB1 from the initial plasmid and pLEX980-hSCG2 was created by replacing the SETDB1 ORF with the one of human SCG2 (NM_003469, SC117954, ORIGENE, Rockville, MD, USA). shRNA plasmid SCG2 KD: TRCN0000055605 (Sigma-Aldrich); pLKO.1-puro non-targeting (NT): #1864 Addgene.

**Real-time PCR**

Total RNA was isolated from cultured cells using the RNeasy Mini Kit (Qiagen, Hilden, Germany), according to manufacturer’s instructions. cDNA was obtained by reverse transcription of 500 ng total RNA using the Revert Aid First Strand cDNA synthesis kit (Thermo Fisher Scientific, Waltham, MA, USA), according to the manufacturer’s instructions. Real time PCR was used to quantify gene expression. For that, cDNA was mixed with SYBR Green Master Mix (Applied Biosystems, Waltham, MA, USA) and specific primers for the gene of interest. All PCR reactions were loaded in triplicates and run on a 7500 Real-Time PCR System device (Applied Biosystems). 18S expression was used as an endogenous control for all experiments. Results were analyzed using the ΔΔC_t_ value method. Analysis was performed using 7500 software, version 2.0.5 (Applied Biosystems).

Primers used (5’-3’ direction):

B2M: GAGGCTATCCAGCGTACTCCA, CGGCAGGCATACTCATCTTTT;

CALR: CCTGCCGTCTACTTCAAGGAG, GAACTTGCCGGAACTGAGAAC;

CANX: CCAAGGTTACTTACAAAGCTCCA, GGCCCGAGACATCAACACA;

SCG2: AGCCGAATGGATCAGTGGAA, GATGGTCTAAGTCAGCCTCTGAGA;

STAT1: ATCCTCGAGAGCTGTCTA, GCCAGGTACTGTCTGATT;

TAP1: CTGGGGAAGTCACCCTACC, CAGAGGCTCCCGAGTTTGTG;

TAP2: CACCTACACCATGTCTCGAATC, AGTTACTCATCAGGGTGGTATCC;

TAPBP: TGGACCGGAAATGGGACC, CCCCAGAAGGGTAGAAGTGG;

18S: GAGGATGAGGTGGAACGTGT, TCTTCAGTCGCTCCAGGTCT.

**Western blot**

For protein extraction, cultured cells were harvested and lysed in RIPA buffer (Sigma-Aldrich) containing 1x Complete Mini Protease Inhibitor Cocktail (Roche) and 1x PhosphoSTOP (Roche). Protein concentrations were determined using Pierce BCA Protein Assay Kit (Thermo Fisher Scientific), according to the manufacturers’ protocol. To separate proteins by size through electrophoresis, 20-30 µg of whole cell lysate were loaded and run on NuPAGE^TM^Novex^TM^ 4-12% Bis-Tris Protein gels (Thermo Fisher Scientific). Afterwards, proteins were transferred onto PVDF membranes (Merck Millipore, Burlington, MA, USA). After transfer, membranes were incubated in a blocking solution for 1h at RT and then incubated over night at 4°C with anti-SCG2 (1:1000; GeneTex, Irvine, CA, USA; GTX54665), anti-Stat1 (1:1000; Cell Signaling Technology, Danvers, MA, USA; D1K9Y; #14994), anti‑pStat1 (1:1000; Cell Signaling Technology; Y701 (D4A7); #7649), anti-calnexin (1:1000; Cell Signaling Technology; C5C9; #2679), anti-TAP2 (1:1000; Cell Signaling Technology; #12259), anti-TAP1 (1:1000; Cell Signaling Technology; E4T4F; #49671), anti-calreticulin (1:1000; Cell Signaling Technology; D3E6 XP; #12238), anti-tapasin (1:1000; Cell SignalingTechnology; E6P2Z XP; #66382), anti-β2-microglobulin (1:1000; Cell Signaling Technology; D8P1H; #12851), or anti-GAPDH (1:10000; Cell Signaling Technology; 14C10; #2118) antibodies, diluted in the same blocking buffer. The next day, membranes were washed with 1x TBST buffer and then incubated for 1h at RT with the suitable HRP-conjungated secondary antibody (1:10000; anti-mouse immunoglobulin G [IgG] HRP-linked antibody, Cell Signaling Technology, 7076; anti-rabbit IgG HRP-linked antibody, Cell Signaling Technology, 7074) diluted in the blocking solution. After additional washing, membranes were exposed to Luminata Forte western HRP substrate (Merck Millipore) and visualized with the ChemiDoc^TM^Touch Imaging System (BioRad, Hercules, CA, USA) and analyzed by ImageJ software (NIH) and Image Lab (BioRad).

**Tissue microarray (TMA) analysis**

Human tissue samples obtained from healthy donors and melanoma patients were processed to TMA samples as previously described [^1^](#_ENREF_1) and stained with anti-SCG2 (GeneTex; GTX54665) antibody. S100β was used as a positive control. TMAs were scanned by the National Center for Tumor Diseases (NCT)-Gewebebank facility, Pathology Unit (Heidelberg, Germany). Two independent persons analyzed the images using the immunohistochemistry score system (score range: 1-12)[^1^](#_ENREF_1). All patients included in this study agreed to the declaration of consent according to the ethical votes 2010-318N-MA and 2014-835R-MA (ethics committee II of Heidelberg University, Germany).

**Flow cytometry**

Cultured cells were harvested and washed with FACS buffer (1x PBS, 0.5% BSA, 0.05% sodium azide). Then, cells were incubated for 1h with APC-conjugated anti-human HLA-A, B, C antibody (#311410, Biolegend, San Diego, CA, USA) at 4°C. After washing the cells, DAPI (Roche) was added to distinguish live and dead cells and samples were analyzed by flow cytometry using a FacsCanto II (BD Biosciences, USA). FlowJo cell analysis software 10.8.1 (BD Biosciences, Franklin Lakes, NJ, USA) was used for data analysis.

**Cell cycle analysis**

Cultured cells were harvested and washed once with ice cold PBS. Afterwards, cells were resuspended in PBS and fixed by adding ice cold 70% ethanol to the PBS. Ethanol was added dropwise while vortexing and afterwards samples were stored over night at 4°C. The next day, PBS was added to the samples and supernatant was removed after centrifugation. Then, samples were incubated 20 min with 0.5 mg/ml RNase A (diluted in PBS) at 37°C. Lastly, PI was added at a concentration of 40 µg/ml followed by incubating 30 min in the dark at RT. Samples were analyzed by flow cytometry using an LSR Fortessa HTS (BD Biosciences). Afterwards, data were assessed using FlowJo cell analysis software 10.8.1 (BD Biosciences).

**Microarray gene expression analysis**

For microarray analysis, RNA samples of SCG2-overexpressing and empty vector control cells were analyzed with the Affymetrix GeneChips® Clariom^TM^ S Assay, human (Affymetrix, Santa Clara, CA, USA) by the Genomics and Proteomics Core Facility at the DKFZ. Gene expression analysis was conducted by the biostatistics division at DKFZ, Germany. Affymetrix CEL files were RMA normalized and expression values log2-transformed. Differentially expressed probesets/genes between groups were identified using the empirical Bayes approach based on moderated t-statistics as implemented in the Bioconductor package limma[^2^](#_ENREF_2)^,^ [^3^](#_ENREF_3). Gene set enrichment analysis was performed using the camera test[^4^](#_ENREF_4). KEGG, Reactome, and gene ontology data bases were used in pathways analysis[^5-7^](#_ENREF_5). All p-values were adjusted for multiple testing using the Benjamini-Hochberg correction in order to control the false discovery rate (FDR). All analyses were performed with statistical software R 4.0.

**T cell cytotoxicity assay**

10.000 melanoma cells were seeded per well on an xCELLigence E-Plate 96 PET (Agilent Technologies, Santa Clara, CA, USA). The plate was incubated in an xCELLigence RTCA MP working station (ACEA Biosciences, San Diego, CA, USA) located in an incubator. 24h later, 100.000 MART-1-specific T cells were added to the cells and killing was recorded by measuring the change in the impedance of the plates. T cells were provided by the Joint Immunotherapeutics Laboratory, German Cancer Research Center (DKFZ), Heidelberg, Germany, generated according to Johnson et al.[^8^](#_ENREF_8). After 36h, recording was stopped and data were analyzed using Prism 5.0 software (GraphPad). xCELLigence RTCA MP working station was operated using the xCELLigence RTCA MP analyzer (ACEA Biosciences). ​

**Data set analysis**

We analyzed correlation between SCG2 and HLA-A, HLA-B, and HLA-C mRNA expression levels in melanoma as well as differences in SCG2 expression levels between normal skin, primary melanoma, and melanoma metastases from the GSE database (GSE7553)[^9^](#_ENREF_9). These mRNA expression levels were provided by R2 Genomics analysis and visualization platform (<http://hgserver1.amc.nl>) accessed on February 11^th^ 2022. Kaplan-Meier curve showing patient survival was generated from available data from DFCI, Nature Medicine 2019 ([www.cbioportal.org](http://www.cbioportal.org)) accessed August 31^st^ 2021[^10^](#_ENREF_10).

**Statistical analysis**

All experiments shown were performed at least in triplicates. Data were analyzed using Prism 5.0 software (GraphPad) and represented as mean ± SEM. To compare two conditions a two-tailed Student’s t test was used, whereas one-way ANOVA was applied to compare multiple conditions. Spearman correlation was used to determine the correlation of two parameters and Kaplan-Meier method was applied for analysis of survival. Statistical significance is indicated with the p-value scale (*p < 0.05; **p < 0.01; ***p < 0.001; “ns” refers to p ≥ 0.05).

**References**

1. Wagner NB, Weide B, Reith M, Tarnanidis K, Kehrel C, Lichtenberger R, et al. Diminished levels of the soluble form of RAGE are related to poor survival in malignant melanoma. Int J Cancer. 2015;137(11):2607-17.

2. Smyth GK. Linear models and empirical bayes methods for assessing differential expression in microarray experiments. Stat Appl Genet Mol Biol. 2004;3:Article3.

3. Smyth GK. limma: Linear Models for Microarray Data. In: Gentleman R, Carey VJ, Huber W, Irizarry RA, Dudoit S, editors. Bioinformatics and Computational Biology Solutions Using R and Bioconductor. New York, NY: Springer New York; 2005. p. 397-420.

4. Wu D, Smyth GK. Camera: a competitive gene set test accounting for inter-gene correlation. Nucleic Acids Res. 2012;40(17):e133.

5. Kanehisa M, Goto S. KEGG: kyoto encyclopedia of genes and genomes. Nucleic Acids Res. 2000;28(1):27-30.

6. Fabregat A, Jupe S, Matthews L, Sidiropoulos K, Gillespie M, Garapati P, et al. The Reactome Pathway Knowledgebase. Nucleic Acids Res. 2018;46(D1):D649-D55.

7. The Gene Ontology Consortium. Expansion of the Gene Ontology knowledgebase and resources. Nucleic Acids Res. 2017;45(D1):D331-D8.

8. Johnson LA, Heemskerk B, Powell DJ, Jr., Cohen CJ, Morgan RA, Dudley ME, et al. Gene transfer of tumor-reactive TCR confers both high avidity and tumor reactivity to nonreactive peripheral blood mononuclear cells and tumor-infiltrating lymphocytes. J Immunol. 2006;177(9):6548-59.

9. Riker AI, Enkemann SA, Fodstad O, Liu S, Ren S, Morris C, et al. The gene expression profiles of primary and metastatic melanoma yields a transition point of tumor progression and metastasis. BMC Med Genomics. 2008;1:13.

10. Liu D, Schilling B, Liu D, Sucker A, Livingstone E, Jerby-Arnon L, et al. Integrative molecular and clinical modeling of clinical outcomes to PD1 blockade in patients with metastatic melanoma. Nat Med. 2019;25(12):1916-27.
